# Amygdala, pulvinar, and inferior parietal cortex contribute to early processing of faces without awareness

**DOI:** 10.3389/fnhum.2013.00241

**Published:** 2013-06-06

**Authors:** Vanessa Troiani, Robert T. Schultz

**Affiliations:** ^1^Department of Neuroscience, University of Pennsylvania School of MedicinePhiladelphia, PA, USA; ^2^Center for Autism Research, Children's Hospital of PhiladelphiaPhiladelphia, PA, USA; ^3^Department of Psychiatry and Pediatrics, University of Pennsylvania School of MedicinePhiladelphia, PA, USA

**Keywords:** fMRI, continuous flash suppression, adolescents, motivated attention, vision

## Abstract

The goals of the present study were 2-fold. First, we wished to investigate the neural correlates of stimulus-driven processing of stimuli strongly suppressed from awareness and in the absence of top-down influences. We accomplished this using a novel approach in which participants performed an orthogonal task atop a flash suppression noise image to prevent top-down search. Second, we wished to investigate the extent to which amygdala responses differentiate between suppressed stimuli (fearful faces and houses) based on their motivational relevance. Using continuous flash suppression (CFS) in conjunction with fMRI, we presented fearful faces, houses, and a no stimulus control to one eye while participants performed an orthogonal task that appeared atop the flashing Mondrian image presented to the opposite eye. In 29 adolescents, we show activation in subcortical regions, including the superior colliculus, amygdala, thalamus, and hippocampus for suppressed objects (fearful faces and houses) compared to a no stimulus control. Suppressed stimuli showed less activation compared to a no stimulus control in early visual cortex (EVC), indicating that object information was being suppressed from this region. Additionally, we find no activation in regions associated with conscious processing of these percepts (fusiform gyrus and/or parahippocampal cortex) as assessed by mean activations and multi-voxel patterns. A psychophysiological interaction analysis (PPI) that seeded the amygdala showed task-specific (fearful faces greater than houses) modulation of right pulvinar and left inferior parietal cortex. Taken together, our results support a role for the amygdala in stimulus-driven attentional guidance toward objects of relevance and a potential mechanism for successful suppression of rivalrous stimuli.

## Introduction

We are automatically drawn to objects that are relevant to our needs and desires. For example, as human beings, we tend to pay more attention to faces and bodies compared to other objects. Emotional stimuli are also processed earlier in this object-relevance hierarchy, potentially due to selective attention mechanisms that are automatically engaged by emotionally salient objects (Vuilleumier and Schwartz, 2001; Vuilleumier, 2005). These category-based preferences are thought to relate to the stimulus meaning or value: Conspecifics are valuable to us due to the important information faces can convey. Emotional stimuli indicate a potential threat, which is meaningful in terms of survival (Ekman and Friesen, 1971; LeDoux, 1998; Öhman et al., 2000; Anderson and Phelps, 2001). Object relevance is also state-dependent: food stimuli are captured by attention more quickly when we're hungry than when we're sated (Mohanty et al., 2008). While it is clear stimulus meaning and motivational value modulate object-based prioritization, it is not fully understood how highly relevant objects are prioritized in attention and how this process is reflected in the human brain.

Meaningful stimuli benefit from enhanced attentional capture, even prior to awareness. In the past, implicit processing of objects was studied using backward masking and binocular rivalry techniques. More recently, a more potent suppression technique has been developed, that of continuous flash suppression (CFS) (Tsuchiya and Koch, 2005). CFS uses rapidly flashing colored images (Mondrians) presented to one eye to prevent awareness of a stimulus presented to the opposite eye. One behavioral use of this technique is the break from CFS paradigm (b-CFS), in which the relevance of a target is determined based on the time it takes to break through the flashing stimulus and reach awareness (Jiang et al., 2007). Using this technique, it has been shown that social signals are prioritized more quickly. For example, observers become aware of a face with a direct gaze faster than one with indirect gaze and upright conspecifics faster than an inverted visual control (Stein et al., 2011, 2012). Stimuli that contain both social *and* emotional signals, like fearful faces, are a particularly potent stimulus. Observers become aware of fearful faces much more quickly than a non-social visual control (houses) and emotional faces break through faster than non-emotional faces (Yang et al., 2007; Troiani et al., 2012). These differences in stimulus break through are thought to reflect enhanced processing that occurs prior to stimulus awareness.

The amygdala plays a particularly important role in spontaneous orienting toward salient parts of a stimulus (such as the eye region of a face) and is thought to facilitate enhanced processing of biologically-relevant stimuli prior to awareness (Whalen et al., 1998, 2004; Adolphs et al., 2005; Adolphs, 2008, 2010; Pessoa, 2010). Patients lacking bilateral amygdalae suffer from impaired automatic orientation toward the salient portions of a face, potentially due to impaired stimulus-driven attention (Tsuchiya et al., 2009; Kennedy and Adolphs, 2010). Connections between the amygdala, the pulvinar nucleus of the thalamus, and the superior colliculus are thought to form an alternate visual pathway that bypasses cortex to provide fast yet coarse visual information with the potential to aid in threat detection (Johnson, 1990, 2005; Morris et al., 1996, 1999). However, due to the profuse interconnections present between the regions of the hypothesized colliculus-pulvinar-amygdala pathway and cortex, it is difficult to limit processing exclusively to these regions. An alternative hypothesis is that the pulvinar and amygdala serve to coordinate the function of cortical networks in the process of evaluating the biological significance of a stimulus (Pessoa and Adolphs, 2010). Under this framework, the cortex remains significantly involved in this process, and processing is not limited to the three regions of the subcortical pathway.

Consistent with the idea of amygdala and cortical involvement in the evaluation of important stimuli, recent work has shown enhanced processing of motivationally relevant stimuli to be reliant on a combination of highly interactive cortical and subcortical structures. It may be the case that it is not merely the involvement of specific brain regions in emotional and motivational processes, but the enhanced communication between cortical and subcortical regions induced by motivational states (Kinnison et al., 2012). Mohanty et al. (2008) showed that following a period of food and water fasting, participants activated a network of regions involved in spatial attention in response to donuts (a food) compared to hexnuts (a visually similar tool). This network included posterior parietal cortex, intraparietal sulcus, frontal eye fields, posterior cingulate, and the amygdala. We recently found a similar network of activation for suppressed motivationally relevant faces compared to a suppressed non-social stimulus (houses) (Troiani et al., 2012). In our previous study, we successfully implemented a novel paradigm designed to limit top-down influences in order to measure stimulus-driven components of object prioritization. In this paradigm, participants perform a demanding task that is unrelated to the suppressed stimuli, which serves to increase the duration of suppression due to a higher perceptual load (Bahrami et al., 2008). This allows for the examination of stimulus-driven neural responses to suppressed stimuli in the absence of top-down search strategies. Here, we combine CFS with a demanding task that appeared atop the flashing Mondrian images in order to suppress images from awareness for the duration of the fMRI study. We further optimized this method in order to increase the depth of stimulus suppression by (1) using a more robust form of suppression, (2) making stimuli smaller to prevent piecemeal breakthrough, (3) using MR compatible goggles with a dual LCD display to prevent escape of certain wavelengths from the suppressed stimulus into the dominant eye, and (4) adding a no-stimulus control condition. With these optimizations, we hoped to strengthen stimulus suppression in order to isolate the earliest regions of the network that contributes to the differential prioritization of stimuli prior to awareness.

## Materials and methods

### Subjects

Twenty-nine adolescents [2 females; ages 11–17 years (mean = 14.3); 2 left-handed] with normal or corrected-to-normal vision were recruited from the Philadelphia community to participate in the main fMRI experiment. All participants gave written informed consent in accordance with procedures approved by the Children's Hospital of Philadelphia Institutional Review Board and were paid for their participation. Prior to the fMRI session, subjects completed a mock scan procedure, allowing the participants to acclimate to the scanner environment and train to minimize movement while scanning. Only participants who were under a minimum movement criterion preceded to the scanning session. None of the participants moved more than 3 mm during any scanning run. Three subjects were eliminated from the connectivity analysis because they did not show activation within the region of interest used to define the seed region.

#### Piloting

In order to establish the effectiveness of the method, six pilot subjects also completed the task while undergoing fMRI. Pilot subjects were six adults (all female) with knowledge of the suppressed stimuli and the goal of the study. The objective of piloting was to determine whether participants with knowledge of the stimuli experienced break from interocular suppression while performing the task. None of the pilot subjects experienced breakthrough of the suppressed stimuli while performing the task. Even when these participants had knowledge of the presence of the suppressed stimuli, they experienced no breakthrough, indicating the effectiveness of this suppression method.

### Magnetic resonance imaging acquisition

Imaging data were collected using a 3T Siemens Verio scanner and a 12-channel head coil. Two structural MR images were acquired for the registration of fMRI data to standard space: a high-resolution T1-weighted MPRAGE sequence of the entire brain (176 sagittal slices, isotropic voxel size = 1 mm, TR = 1900 ms, TE = 2.54 ms, flip angle = 9°), and a high-resolution FLASH sequence collected in the same axial plane as the fMRI data (number of slices = 40, slice thickness = 3.5 mm, TR = 300 ms, TE = 2.46 ms, flip angle = 60°). Functional data consisted of two 4-minute runs of whole-brain T2^*^ weighted BOLD echoplanar images with 107 volumes acquired per run (40 oblique axial slices, isotropic voxel size = 3.5 mm, TR = 2340 ms, TE = 25 ms, flip angle = 90°).

### Stimuli

Stimuli of interest were 32 gray scale fearful faces and 32 houses presented within 2° of visual angle into the left lens of MR compatible dual display LCD goggles (Resonance Technology Inc., Northridge, CA). Responses were recorded with a four-key fiber optic response box. Task stimuli consisted of movies of colorful Mondrian images changing at a rate of 10 Hz. Mondrian images were created using Matlab, with each 28-s block movie consisting of 280 unique dynamic noise images, each presented for 100 ms. Images were made into movies using Corel Video, with letters and fixation cross images added to these movies before exporting the movies to Quicktime. Experimental presentation was done with Psychopy (Peirce, 2007, 2009).

**Figure 1:**
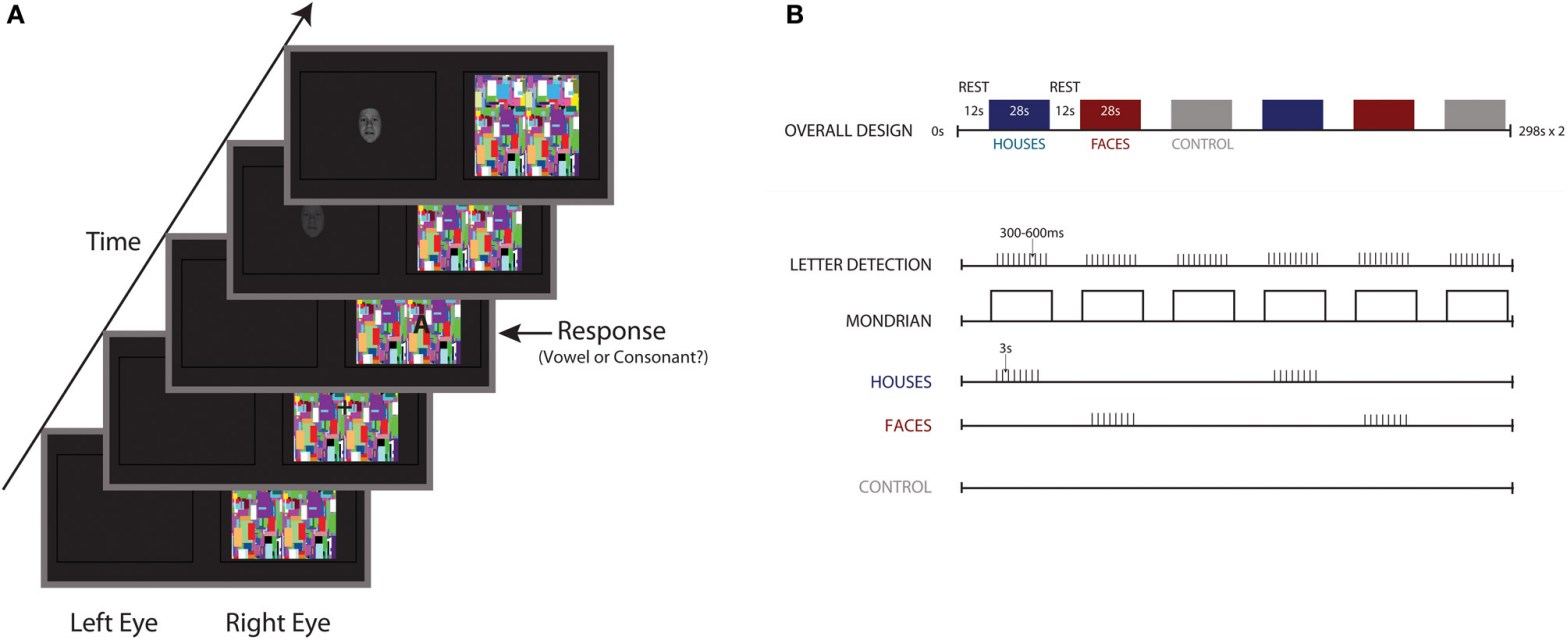
**Stimulus schematic and experimental design. (A)** Participants performed a vowel/consonant detection task, projected into the right eye atop flashing noise images presented at a rate of 10 Hz. In the left eye, 32 fearful faces, 32 houses, and a no stimulus control were projected to the left eye. **(B)** Overall block design, with 28-s blocks of noise images separated by 12 s of rest. Ten letters were presented for a duration of 1500 ms each, with letter onset jittered by 300–600 ms. Eight houses or fearful faces appeared within each block, with block order counterbalanced and randomized across participants.

A fixation cross appeared in the center of the Mondrian movies, and uppercase letters from the English alphabet appeared in one of four quadrants immediately adjacent to the fixation cross. Letters consisted of 5 vowels (A, E, I, O, U) and 5 consonants (C, H, N, T, S). The task consisted of 12 28-s blocks (12 TRs, 2340 ms each TR). Within a block, letter trials appeared in the right eye and stimulus trails to the left, which was experienced by the subject as one image (see Figure 1A). Following is a description of these trials as incorporated into a block (For visual schematic, see Figure 1B). Each trial was a total of 2340 ms, the length of one TR. Projected through the right lens, a block began with a continuous stream of Mondrian images changing at a rate of 10 Hz. After 2340 ms, the first of 10 letter trials was presented. A letter trial consisted of a 300 ms fixation cross, followed by the appearance of a letter in one of the four quadrants for a duration of 1500 ms. Onset of the letter trials was varied by 300–600 ms from the start, with the difference in onset accounted for at the end of a trial, such that each letter trial was 2340 ms. At the end of 10 trials, only the Mondrians appeared for 2340 ms (no letters or fixation) and then the block was complete. In each block, all 10 letters were presented, with letter order and onset variance randomized between blocks.

Stimuli of interest were projected through the left lens, blocked by stimulus category, with category order counterbalanced across participants. Eight fearful faces, eight houses, or a no-stimulus control were presented in each block. A block began with a black screen for the first 4680 ms. After this period, 8 stimulus trials were presented. A stimulus trial began with a stimulus that appeared after 600 ms. The stimulus was slowly ramped from a contrast level of 0 to 100 over 750 ms and ramped back down over the following 750 ms (total duration: 1500 ms). The left screen was then blank for another 340 ms until another trial began. Following the presentation of 8 trials, no stimulus appeared for another 4680 ms until block completion. Task blocks were separated by 11,700 ms of rest, with a black screen presented to both eyes. It should be noted that for the no stimulus control condition, a black screen was presented to the left eye for the entire 28-s block, while the task still appeared in the right eye.

### Procedure

The main fMRI experiment consisted of two 4 min 20 s scan runs, each of which was divided into 6 task blocks and 7 periods of rest. During each block, participants viewed letters that appeared surrounding a central fixation. They were instructed to press the right button if the letter was a vowel and the left button if the letter was a consonant. Following the presentation of the 2 runs, a catch trial was presented. A catch trial consists of a fearful face or house image presented atop of the Mondrian image to both eyes, in order to mimic break from interocular suppression. This trial is used as a probe to assess whether participants experienced break from interocular suppression earlier in the experiment. Following the catch trial, participants were asked, “Did you notice anything different about the last 2 trials?” All participants reported the presence of a face and a house. They were then asked if they saw any objects or parts of objects earlier in the experiment. All participants reported that they did not see objects prior to the catch trial, indicating successful suppression of the objects for the duration of the experiment.

Following the main experimental scans, a 5-min functional localizer scan was administered, in which subjects detected when a centrally presented white crosshair appeared on full color faces, scenes, objects, and scrambled objects, presented in a blocked design. Four, 14-s blocks of each image category were presented as “superblocks,” in which the stimulus category blocks were presented in succession and separated by 14 s of rest. Each “superblock” sequence was presented four times, with object categories in a different order for each “superblock.”

### Data analysis

Image preprocessing and statistical analyses were performed using SPM8 (Wellcome Trust Centre for Functional Neuroimaging, London, UK). Functional images from both experimental and localizer scan runs were initially analyzed separately for each participant. Low-frequency drifts were removed with high-pass filtering with a cutoff period of 128 s and autocorrelations modeled using a first-order autoregressive model. Images for each participant were realigned to the first image in the series (Friston et al., 1995) and coregistered with the structural image (Ashburner and Friston, 1997). The transformation required to bring a participant's images into standard MNI152 space were calculated using tissue probability maps and these warping parameters were then applied to all functional images for that participant (Ashburner and Friston, 2005). The data were spatially smoothed with a 4 mm FWHM isotropic Gaussian kernel.

#### Whole brain analysis

Whole-brain analyses were implemented using a standard linear modeling approach. These models included three categorical regressors indicating whether the suppressed stimulus for each block was a fearful face, house, or no stimulus control. Categorical regressors were boxcar functions at stimulus onset convolved with a canonical hemodynamic response function. Whole brain analyses were corrected for multiple comparisons using a cluster corrected family wise error (FWE) threshold of *p* < 0.05.

#### Region of interest analysis: subject's individual parameter estimates

Our main region of interest was the amygdala, based on its involvement in implicit processing of social and emotional stimuli. The amygdala is composed of multiple subnuclei, with each nucleus displaying different response profiles and structural connectivity. We used the three amygdala sub-regions of the cytoarchitectonic probability maps to explore response profiles to the suppressed image conditions and the no stimulus control (Amunts et al., 2005). For these analyses, average parameter estimates were extracted for each sub-region in both hemispheres using Marsbar (Brett et al., 2002).

We were also interested in responses in ventral visual cortex to the suppressed images. Because of the variance between subjects in object-selective cortex, we defined two functional regions of interest in each subject using data from the functional localizer scans. The fusiform face area (FFA) was defined as the region of the fusiform gyrus responding more to faces than to scenes. The Parahippocampal Place Area (PPA) was defined as the set of contiguous voxels responding more strongly to scenes than objects in the posterior parahippocampal/collateral sulcus region. Significance thresholds (ranging from *t* > 3.0 to *t* > 4.0) were set for each ROI on a subject-by-subject basis. Using these criteria, there was 1 subject with no FFA bilaterally, 2 subjects with no left FFA, 3 subjects with no left PPA, and 3 subjects with no right PPA.

For individual parameter estimate ROI analyses, the time course of response during the main experiment was extracted from each ROI and response estimates (i.e., Beta values) were obtained for each regressor and covariate, which were then compared between conditions using a repeated measures ANOVA with follow-up *t*-tests, when appropriate.

#### Multivoxel pattern analyses

In the FFA and PPA, we performed multivoxel pattern classification in addition to the univariate analyses. This analysis was only done on participants with bilateral FFA and PPA (*N* = 21). Preprocessing for the MVPA analysis was identical, except data were not spatially smoothed. Three regressors were created to model each of the conditions of interest (fearful faces, houses, control) separately within the two experimental runs. After using these regressors to extract beta values for each condition at every voxel, we performed multivoxel pattern classification on these values using custom MATLAB code based on the method described by Haxby et al. (2001). In this analysis, we calculated a cocktail mean pattern for each of the two runs and subtracted this mean from each of the individual patterns prior to classification. Pattern classification was performed by pairwise comparisons across all 3 conditions (fearful faces, houses, and control). If the average pattern correlation between fearful faces in opposite halves of the data was higher than between fearful faces and houses in opposite halves of the data, this was considered a correct classification. Classification accuracy was then averaged across all possible pairwise comparisons for a given ROI and tested against random chance (i.e., 0.5) using a one-tailed *t*-test.

#### Connectivity analysis

In order to examine whether the amygdala increases in coherence with regions of an attention network that we identified previously we employed a psychophysiological interaction analysis (PPI) (Friston et al., 1997). In this analysis, a seed region is identified and the interaction of this seed region and a covariate of interest (in this instance, suppressed Faces > suppressed Houses) is computed. The resultant interaction term is then entered as a covariate in a general linear model, along with additional covariates for the response of the seed region and the covariate of interest. Any significant effects corresponding to the interaction term are thought to reflect increased coherence or functional connectivity with the seed region. We have used this method previously with an amygdala seed and identified a network of activation, including the pulvinar nucleus of the thalamus, insula, frontal eye fields, early visual cortex (EVC), intraparietal sulcus, and frontal eye fields. Subject-specific amygdala peaks were identified as a 4 mm sphere surrounding the maxima within a superficial amygdala ROI, for the suppressed face > suppressed house contrast. For each ROI, the first eigenvariate of the timeseries was extracted to summarize the timecourse of activation. Neural activity was then estimated using a simple deconvolution model; the estimated neural activity was then multiplied by the psychological variable (faces vs. houses) and reconvolved with a canonical HRF to obtain an interaction term. Individual subjects' data were then modeled using the ROI timecourse, psychological variable (i.e., stimulus type: suppressed faces vs. suppressed houses), and interaction term as regressors. Contrast images were created for the interaction term, which reflected correlations between the seed region that differed depending on stimulus category. We then extracted average beta values from each subject for each of 7 regions of interest, based on connectivity results from our previous work (Troiani et al., 2012).

## Results

### Whole brain analysis

**Figure 2:**
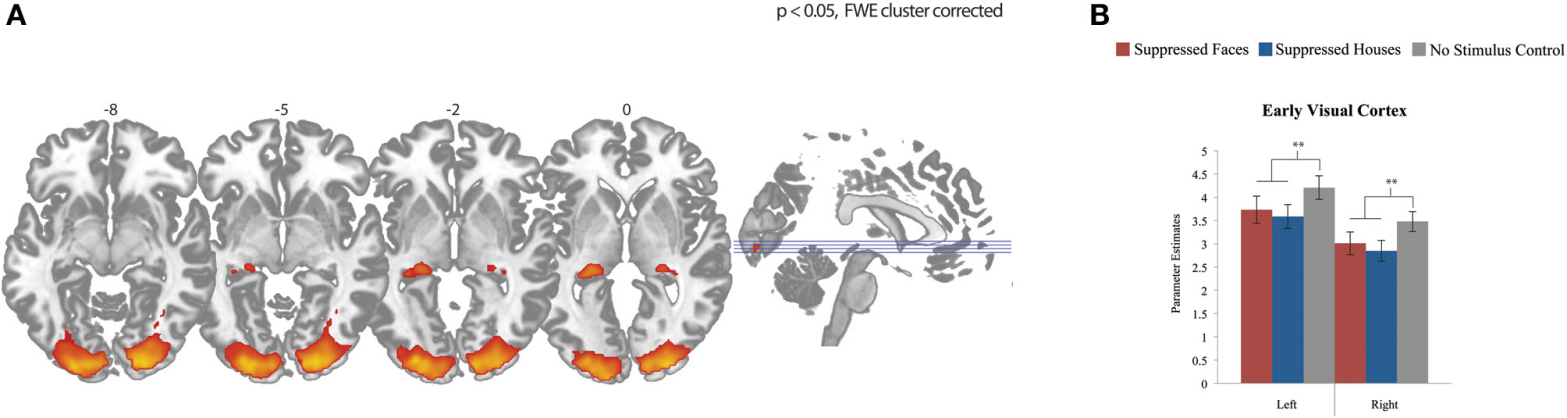
**Effects across all conditions. (A)** Whole brain fMRI response to fearful faces, houses, and control compared to resting baseline. Data show effects in bilateral lateral geniculate nucleus and early visual cortex, FWE cluster corrected for multiple comparisons, *p* < 0.05. **(B)** fMRI response in early visual cortex for each condition, plotted individually by hemisphere. Differences between stimulus presentation (fearful face or house) and no stimulus (control) were significant in both hemispheres, with stronger activation for the control condition than the two stimulus conditions. There were no differences between the two stimulus categories (fearful faces compared to houses).

We first assessed the activation pattern evoked by the conscious task (flashing Mondrian images presented to the right eye). To examine this, we averaged activation across the three covariates (fearful faces, houses, and control) compared to a resting baseline (12 s blocks of rest). Because the Mondrian images are consistent across these three conditions, we expected activation in regions of the central visual system. Indeed, participants activated bilateral lateral geniculate nucleus (LGN) and EVC (Figure 2A; Table 1). We then explored whether there were differences in EVC between three conditions by extracting subject's parameter estimates from each condition, separately, using a mask defined by the regions reaching whole brain significance. (We chose not to explore the LGN signal further, as there is a great deal of anatomical variability in subject anatomy and we would be unable to differentiate the LGN from surrounding structures). We observed a significant effect of condition in EVC, bilaterally [left *F*_(2)_ = 6.83, *p* = 0.002; right *F*_(2)_ = 12.01, *p* < 0.001] (Figure 2B). However, this was driven by stronger activation when there was no stimulus presented to the left eye compared to a fearful face or house stimulus [RIGHT: faces *t*_(28)_ = 3.92, *p* = 0.001; houses *t*_(28)_ = 5.17, *p* < 0.001; LEFT: faces *t*_(28)_ = 2.98, *p* = 0.006; houses *t*_(28)_ = 3.54, *p* = 0.001]. We find no significant differences between fearful face and house conditions in EVC.

**Table 1:** **Peaks of significant clusters for all conditions and for the contrast of stimulus (faces and houses) compared to no stimulus**.

**Contrast**	**Region**	**Hemi-**	***x***	***y***	***z***	***T*-value**
		**sphere**				
All conditions	LGN	R	24	−28	0	6.63
	LGN	L	−26	−32	0	7.53
	EVC	R	24	−96	8	15.31
	EVC	L	14	−102	8	16.1
Stimulus > No stimulus	Amygdala	R	26	−2	−16	3.91
	Thalamus	R	12	−2	0	4.09
	Superior colliculus	R	4	−22	−6	3.60
	Hippocampus	R	28	−12	−10	5.03

**Figure 3:**
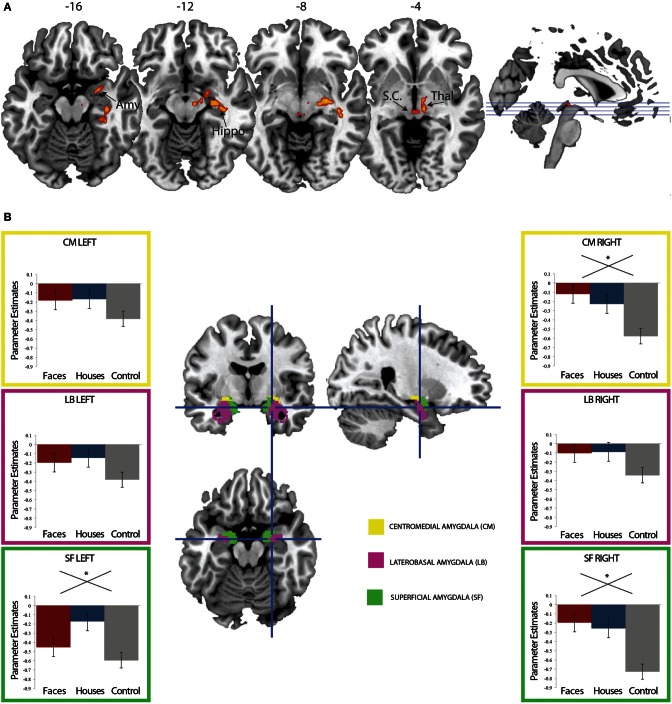
**(A)** Regions showing greater fMRI response to suppressed fearful faces or suppressed houses compared to control. Activation to stimulus (fearful faces and houses) compared no stimulus was computed. Suppressed stimuli activated the right amygdala, superior colliculus, thalamus, and hippocampus compared to the no stimulus control condition (FWE *p* < 0.05 cluster corrected for multiple comparisons). **(B)** Region of interest analysis using amygdala cytoarchitectonic probabilistic maps. An average parameter estimate for the centromedial (yellow), laterobasal, and superficial subregions was computed for each individual across all three conditions. While every subregion show the same pattern of response (stronger response for fearful faces and houses compared to control), this reached significance in bilateral superficial amygdala and the right centromedial amygdala.

Results are cluster FWE corrected for multiple at p < 0.05. Regions correspond with Figures 2A and 3A.

When contrasting the conditions with a stimulus (fearful faces or houses) with the no stimulus control condition, we find a single cluster of activation that encompasses right lateralized superior colliculus, thalamus, amygdala, and hippocampus, can a reference to Figure 3A and Table 1. These results are consistent with an abundance of previous work implicating these regions in implicit perception and vision without awareness (De Gelder et al., 1999; De Gelder and Hadjikhani, 2006; Tamietto et al., 2009; Stienen and De Gelder, 2011; Van den Stock et al., 2011). However, there are no differences between fearful faces and houses based on mean activation in these subcortical regions. Even when we lower this contrast to an excessively liberal threshold (*p* < 0.05, uncorrected), the regions showing mean differences to stimulus vs. no stimulus are only in subcortical areas. Based on our a priori hypothesis regarding the amygdala, we examine responses in this region statistically using a region of interest approach, described below.

### Amygdala response: region of interest with subject's individual parameter estimates

The amygdala is frequently activated by social information and is thought to play a particular role in guiding orientation responses to visual social stimuli (Adolphs and Spezio, 2006; Adolphs, 2008, 2010). We have previously found amygdala activation to fearful faces (an emotional, social stimulus) in the absence of awareness (Pasley et al., 2004; Troiani et al., 2012). Thus, we expected a differentially stronger response in the amygdala for fearful faces compared to houses. We explored this hypothesis by examining responses in bilateral amygdala, for each of three regions defined by cytoarchitectonic probabilistic maps (Amunts et al., 2005). Contrary to our hypothesis, we did not find amygdala activation that was specific to fearful faces. Instead, in all amygdala ROIs, we observed an effect of condition (stimulus vs. no stimulus) in bilateral superficial amygdala and the right centromedial amygdala [Left SF: *F*_(2)_ = 3.18, *p* = 0.049; Right SF: *F*_(2)_ = 7.15, *p* = 0.002; Right CM: *F*_(2)_ = 6.74, *p* = 0.002], but there were no differences in activation between fearful faces and houses (Figure 3B). Please note that these are relative differences in activation, such that in the control condition, the amygdala is quite suppressed compared to baseline. The amygdala is known to undergo suppression compared to a resting baseline during an attention-demanding task (such as detecting letters in a noise pattern) (Morawetz et al., 2010; Stjepanovic et al., 2011). Thus, we interpret the less negative amygdala response to fearful face and house stimuli as a small break from the suppression of the amygdala. While we did not observe a category-specific response in the amygdala to fearful faces, we go on to explore the connectivity profile of the right superficial amygdala, based on its involvement in social processing (Goossens et al., 2009; Bos et al., 2013; Bzdok et al., 2012).

### Amygdala connectivity analysis

**Figure 4:**
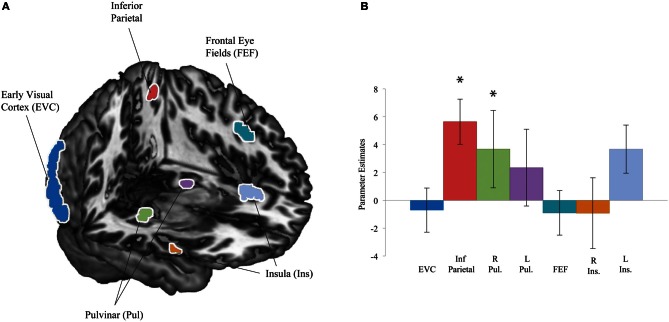
**Connectivity regions of interest and results. (A)** Regions of interest were defined based on a result from our previous work: regions that showed increased coherence with the amygdala for fearful faces compared to houses included early visual cortex (EVC), left inferior parietal cortex, bilateral pulvinar (Pul), left frontal eye fields (FEF), and bilateral insula (Ins) (Troiani et al., 2012). **(B)** Region of interest results from a psychophysiological interaction (PPI) analysis with a right superficial amygdala seed. Significant effects were observed in left inferior parietal cortex (red) and the right pulvinar (green). ^*^p < 0.05

We previously identified a network of increased coherence with the left amygdala BOLD signal for suppressed fearful face presentations compared to suppressed houses (Troiani et al., 2012). One goal of the current study was to examine whether this network existed with a more robust form of interocular suppression. Based on our finding of right superficial amygdala activation to both faces and houses, we used this region to guide a connectivity analysis. We reasoned that despite the lack of differential mean activation in this region based on the category of the stimulus, perhaps this activation leads to increased connectivity for one stimulus (fearful faces) more than another (houses), based on its motivational value. We used regions of interest from the results of our previous connectivity analysis to guide our search. These seven ROIs included bilateral pulvinar, bilateral insula, left inferior parietal cortex, left frontal eye fields, and EVC (Figure 4A). We find increased coherence between the right superficial amygdala seed and two regions, including the right pulvinar and left inferior parietal cortex (Figure 4B). These results suggest that the pulvinar and parietal cortex may be amongst the earliest regions to differentiate between motivational stimuli, a point we will take up further in the discussion.

### Univariate ventral visual responses

The FFA and PPA are regions typically defined based on their category-selectivity (Kanwisher et al., 1997; Epstein and Kanwisher, 1998). In conscious vision, the FFA responds most strongly to faces compared to other objects, while the PPA responds most robustly to scenes or houses and not at all to faces. In studies of non-conscious vision, activation in category specific regions is thought to reflect stimulus awareness, as activation in these regions may indicate that the signal from the visual stimulus has escaped suppression enough to proceed beyond early regions in the visual processing hierarchy and reach higher level processing regions. Although, some studies have found activation in category-specific visual cortex without awareness albeit at much lower levels compared to responses to conscious stimuli (Jiang and He, 2006; Troiani et al., 2012). Given the link between conscious awareness and activation in category-selective cortex, we examine mean responses in the FFA and PPA to all three conditions (fearful faces, houses, no stimulus control). We find no differences between the three conditions in either FFA [*F*_(2, 25)_ = 0.63, *p* = 0.54, *n.s*.] or PPA [*F*_(2, 25)_ = 1.92, *p* = 0.160, *n.s*.], indicating that the stimuli are not escaping suppression enough to reach ventral visual cortex.

### Multivariate ventral visual responses

A previous study found that faces and houses presented in the absence of awareness were associated with distinct multi-voxel patterns in object-selective cortices (Sterzer et al., 2008). These results suggested that some amount of information escapes suppression and reaches object-selective cortex differentially by object type (i.e., FFA for faces and PPA for houses). To examine whether object-related information was present in our own data, we employed a multi-voxel pattern analysis. We find no evidence that signals in subject-specific FFA or PPA are able to discriminate between fearful faces, houses, or control [FFA left: *t*_(20)_ = 1.32, *p* = 0.203; FFA right: *t*_(20)_ = 0.93, *p* = 0.365; PPA left: *t*_(20)_ = 1.09, *p* = 0.288; PPA right: *t*_(20)_ = 0.992, *p* = 0.332]. In conjunction with the null univariate results described above, these results suggest that stimulus information does not escape suppression enough to reach higher-level cortex in the current experiment.

## Discussion

The goal of this study was to capture stimulus-driven activation that is uncontaminated by top-down mechanisms. We achieve this goal by using an interocular suppression technique accompanied by an orthogonal task that appears atop the dominant image—this task serves to further prevent perception of the stimuli presented to the opposite eye. We successfully implemented this novel paradigm in previous work (Troiani et al., 2012), but optimized the current design by (1) using a more robust version of interocular suppression, (2) making stimuli smaller to prevent breakthrough, (3) using MR compatible dual-display goggles to ensure stimuli were uniquely presented to one eye, and (4) including a no stimulus control condition. Despite the strong suppression that resulted from these optimizations, we find that stimuli (compared to a no stimulus control) can escape interocular suppression and activate regions involved in subcortical vision, including the superior colliculus, thalamus, hippocampus, and a region of particular interest—the amygdala.

There are several differences between the current study and our previous study, both in design and results. These differences include the method of suppression (anaglyph glasses vs. dual display goggles), the lack of mean amygdala differences between object categories in the current study, and the lack of mean activation differences in object-selective ventral visual cortex. We review these differences below and discuss potential reasons for these discrepancies, as well as the implication of these differences on understanding processing of stimuli outside of awareness.

### Differences in method of suppression

Although the combination of flash suppression and rivalry used in our previous study (Troiani et al., 2012) is referenced as a form of CFS, there are a few, important differences. In our previous design, we used a single red/blue rivalrous image that was viewed through anaglyph glasses. Because it is difficult to exactly match the colored lenses of the anaglyph glasses and the color of the rivalrous stimuli, it is possible that certain wavelengths can “leak through” from the suppressed image into the dominant eye. Here, we used MR compatible goggles with a dual LCD display, which allowed for stimulus presentation directly into one eye without the possibility of wavelength-based “leak through” of information. Previously, we induced motion suppression through the use of a centrally presented word/checkerboard stimulus that moved around the screen. In practice, this was quite suppressive—and participants were still not explicitly aware of the stimuli. However, completely changing a colorful, dominant stimulus at a rapid rate [as in the type of CFS described by Tsuchiya and Koch (2005)] is a much stronger form of suppression. Indeed, there are published reports indicating that not all forms of rendering a stimulus unconscious produce similar results. Although backward masking (BM) has been used for years to render stimuli invisible, the use of backward masking and CFS in otherwise identical paradigms produce different results. In a behavioral study using BM in one experiment and CFS in another, non-conscious affective priming was achieved for both happy and angry faces in the BM study and only for angry faces using CFS (Almeida et al., 2013). Thus, it appears that using the less robust BM technique, more information is processed despite equivalent phenomenological suppression. CFS can also be implemented in multiple ways. One method is to use a spectrally separated image and anaglyph glasses. Another is to present completely independent inputs: this can be done by separating the eyes with a piece of cardboard and using prism goggles or using a dual display head mounted device that presents separate images to each eye. Although the suppression strength of (1) CFS using a single, spectrally separated image and anaglyph glasses, and (2) CFS using independent visual inputs has not been explicitly compared using the same paradigm, findings from previous work also suggests potential differences between these implementations. Fang and He (2005) used anaglyph glasses and spectrally separated images in a CFS paradigm to demonstrate that categorically-distinct information can reach the dorsal stream but not the ventral stream. Furthermore, images of suppressed tools evoked stronger dorsal stream activation than suppressed faces. However, Caplovitz et al. (2010) fail to replicate this effect using a CFS experiment with independent displays. One possible explanation for these discrepancies is that CFS implemented with spectral images and anaglyph glasses is a less robust form of suppression than CFS implemented with completely independent visual inputs (due to wavelength-based “leak through” of information from one image to the opposite eye). Similar to the reported differences in suppression strength between BM and CFS, we suggest there may also be differences between using CFS-induced suppression with anaglyph glasses and a single, spectrally altered image compared to CFS-induced suppression with completely independent visual inputs.

### Differences in mean amygdala activation

In our previous study, stimuli could be differentiated based on mean amygdala activation. That is, unseen fearful faces activated more amygdala than unseen houses. Accompanying this greater amygdala activation was left parietal activation for suppressed fearful faces compared to suppressed houses and increased connectivity with multiple regions involved in attention. Thus, we expected to replicate our previous finding of category-specific activation in the current study, despite employing several methods to further prevent escape from suppression. In contrast to our hypothesis, we show equally robust amygdala activation to both fearful faces and houses presented outside of awareness. At the whole brain level, both suppressed stimulus categories activated the right superficial amygdala, a result that was confirmed with a more thorough analysis of amygdala subregions.

In our previous work, we also identified a network of region that increased in coherence with the amygdala for fearful faces compared to houses (pulvinar, insula, inferior parietal, frontal eye fields, and EVC). We partially replicate this finding in the current study: the amygdala connectivity profile showed differential increases in connectivity for suppressed fearful faces compared to suppressed houses. Specifically, we find increased task-specific coherence between the amygdala and two regions that are part of the attention network identified in our previous work: the right pulvinar nucleus of the thalamus and left inferior parietal cortex. Taking the results of both studies together, we speculate that under the less robust suppression induced previously, more information was able to escape suppression and activate a broader network involved in preattentive stimulus processing. With this greater information breaking through, feedforward, and feedback signals between regions in this network may strengthen their communication and lead to the mean activation differences observed in our previous study.

### Differences in object selective cortex

We also examined mean activation and multivoxel pattern differences in cortical regions associated with category-specific processing of faces (FFA) and houses (PPA). In our previous study, we found that fearful face-specific amygdala activation was accompanied by activation in the left FFA, but no activation in PPA for either suppressed faces or houses. In the current study, we find no category-specific activations to the suppressed stimuli. Furthermore, there seems to be no information at all about the presence of a stimulus in high-level visual cortex, as there were no activation differences in either FFA or PPA for the presence of a suppressed stimulus vs. no stimulus. Additionally, neither the FFA nor PPA could discriminate between the presence of a stimulus vs. no stimulus based on multi-voxel patterns, providing further evidence that stimulus information was not reaching high-level visual cortex and indicating that these stimuli were robustly suppressed from awareness.

### Regions involved in processing suppressed stimuli

Because we additionally included a no stimulus control condition in the current design, we were also able to compare activity in visual processing regions in response to the presentation of a suppressed stimulus compared to no stimulus. Unsurprisingly, we show that the main task activates bilateral LGN and EVC, consistent with information processing by a retino-geniculate-cortical pathway. When further exploring activation in EVC to each condition separately, we find significant differences between stimulus presentation and control. More specifically, the control condition correlated with more activation in EVC than the two suppressed stimulus conditions. V1 is the first stage in the visual processing hierarchy at which the information from both eyes is combined. In previous studies examining the neural bases of binocular rivalry, activation in V1 has been concomitant with awareness. That is, when subjects were asked to report whether they perceived one rivalrous stimulus compared to another, activation in V1 strongly correlated with the reported percept (Polonsky et al., 2000; Tong and Engel, 2001; Lee et al., 2007). When stimuli are reliably suppressed, this is associated with suppression in V1 (Lee and Blake, 2002). Because observers remained unaware of the stimuli presented to their left eye for the duration of the study, this pattern of activation in EVC likely reflects successful suppression of the fearful face and house stimuli.

These results are also informative with regard to the idea of parallel visual pathways. Visual signals originate from the retina and project to the LGN and onto the primary visual cortex (V1), located in the posterior occipital lobe, surrounding the calcarine fissure. It is thought that a parallel pathway exists which projects from the superior colliculli to the thalamus, and onto the amygdala. In our data, we show that the presence of a stimulus appears to reduce activation in EVC. In contrast, we show that stimulus information activates regions of the superior colliculus, thalamus, hippocampus, and amygdala, indicating that information has reached structures of the superior colliculus-pulvinar-amygdala pathway. This suggests that information can reach subcortical regions and influence the amygdala without corresponding information representation in higher-level visual regions (FFA/PPA) or even lower level cortical visual regions (EVC).

We also find hippocampal activation when stimuli are present (but suppressed). This finding is consistent with models of fear conditioning that implicate hippocampal-amygdala connections in contextual fear conditioning. For example, Alvarez et al. (2008) found right anterior hippocampus and bilateral amygdala activation for the conditioned stimulus in a foot shock fear conditioning paradigm, but only when preceded by the associated context. Amygdala-hippocampal connectivity increases bidirectionally when retrieval of emotional information is relevant to the current behavior (Smith et al., 2006). Furthermore, unseen primes have been shown to generate predictive signals related to stimulus history and influence the percept selected in a binocular rivalry paradigm (Denison et al., 2011). Thus, it may be that predictive signals are generated by the hippocampus even with the minimal amount of information that escapes interocular suppression. Such a predictive signal would aid in the prioritization of particularly relevant stimuli.

## General discussion

Here, we find amygdala activation for stimuli (vs. no stimulus) presented in the absence of awareness despite apparent suppression of EVC and a lack of information in high level category-specific cortices. These results indicate that information can proceed in a feed-forward manner to the amygdala. We additionally show increased connectivity from the right amygdala to the right pulvinar and left inferior parietal cortex. These results suggest that in addition to the amygdala, the pulvinar, and parietal cortex may be amongst the earliest regions to differentiate between motivational stimuli. Recently, these regions have been implicated in information integration and motivational relevance. Parietal cortex has long been associated with spatial attention and has been more recently implicated in housing a salience map that integrates top-down and bottom-up attention (Balan and Gottlieb, 2006; Bendiksby and Platt, 2006; Fecteau and Munoz, 2006; Geng and Mangun, 2009; Zenon et al., 2010). In particular, the lateral intraparietal cortex (LIP) integrates sensory and reward information (Rorie et al., 2010), is modulated by sensory, motivational, and motor factors (Gottlieb et al., 2009), and has sharpened tuning responses in response to motivational relevance (Falkner et al., 2010). Recently, baseline fluctuations in LIP response were found to reflect motivational fluctuations, independent of spatial attention (Wang et al., 2012). The pulvinar nucleus of the thalamus is a second region implicated in modulating information flow in response to altered motivation. Although this region was previously thought to be merely a relay nucleus, recent evidence highlights a role for the pulvinar in selecting salient information, as pulvinar lesions lead to inabilities to filter out distracting information (Snow et al., 2009; Wilke et al., 2010). The pulvinar has also been specifically implicated in processing salient face information, as emotional expressions of human faces activate neurons in the monkey pulvinar (Maior et al., 2010). Most recently, the pulvinar was shown to synchronize activity between multiple cortical areas (Saalmann et al., 2012), highlighting its complex role in information integration that would be necessary for combining the wide array of information important for assessing motivational relevance. Thus, our finding adds to the growing number of studies implicating the amygdala, pulvinar, and parietal cortices in early processing of motivational stimuli.

Relevant stimuli benefit from increased attentional priority, even prior to awareness. The majority of neuroimaging and lesion-based studies of non-conscious emotional vision have focused on the reactivity of the amygdala or tested the existence of a subcortical pathway that responds preferentially to social/emotional stimuli. Increasingly, the neural evidence suggests a more complex network of regions is involved in processing information outside of awareness and ultimately using this information to influence attention, conscious visual processes, and behavior. CFS is a particularly useful method for examination of these questions, particularly for threat-related emotions, like anger or fear. Other authors have suggested that CFS in particular (as compared to backward masking) results in a processing bias toward neural regions involved in fast but course processing (retinotectal route to amygdala, orbitofrontal cortex, or dorsal stream) (Almeida et al., 2013). This crude processing may function to identify regions of interest that signal danger or ambiguity- and trigger appropriate networks for subsequent analysis, attentional regulation, and behavioral modification.

### Conflict of interest statement

The authors declare that the research was conducted in the absence of any commercial or financial relationships that could be construed as a potential conflict of interest.
